# Measuring School Contexts

**DOI:** 10.1177/2332858415613055

**Published:** 2015

**Authors:** Chandra L. Muller

**Affiliations:** Department of Sociology, University of Texas at Austin

**Keywords:** school context, youth development, social relationships

## Abstract

This article describes issues in measuring school contexts with an eye toward understanding students’ experiences and outcomes. I begin with an overview of the conceptual underpinnings related to measuring contexts, briefly describe the initiatives at the National Center for Education Statistics to measure school contexts, and identify possible gaps in those initiatives that if filled could provide valuable new data for researchers. Next, I discuss new approaches and opportunities for measurement, and special considerations related to diverse populations and youth development. I conclude with recommendations for future priorities.

For over half a century, education researchers have recognized the importance of school contexts for many aspects of education, from teaching and learning to the hidden curriculum that reproduces social inequality. Harnessing the prosocial power of school contextual effects on students’ development, either directly or indirectly by enhancing the work of school professionals, is valuable because elements of the school context may be responsive to carefully crafted education policy. However, assessing and determining successful policy depend on adequate data and models to estimate effects. In contrast to the physical or financial resources of a school or district, many of the school contexts that contribute to academic outcomes are inherently social and depend on social relationships, both potential and realized. Furthermore, any particular context may impact different people differently, and students inhabit multiple contexts simultaneously. Consequently, schools have many contexts under one roof, with the social nature of the school context presenting challenges to measurement over time and space.

This article describes issues in measuring school contexts with an eye toward understanding students’ experiences and outcomes. I begin with an overview of the conceptual underpinnings related to measuring contexts, briefly describe the initiatives at the National Center for Education Statistics (NCES) to measure school contexts, and identify possible gaps in those initiatives that if filled could provide valuable new data for researchers. Next, I discuss new approaches and opportunities for measurement, and special considerations related to diverse populations and youth development. I conclude with recommendations for future priorities.

## Students, Schools, and Their Social Contexts

Over the course of childhood and adolescence, students spend many hours in classrooms, extracurricular activities, summer programs, jobs, parent outreach events, and other opportunities for interaction of peers, friends, and their families. These settings typically change as students grow and gain capacity to handle more independent and complex academic challenges and social relationships. Through these ongoing and at times intense interactions from sharing so many hours of the day over the years of childhood and adolescence, the school can emerge as a venue to define a community.

### The Student in the School Context

Beginning with the individual, school community contributes to a student’s identity development. From Cooley’s (1902) classical looking-glass self or a more updated version from the popular 1985 film *The Breakfast Club* (see Barber, Eccles, & Stone, 2001), social scientists have recognized the crucial role of others in shaping identity, and of identity in shaping behavior and other outcomes. Indeed, the notion that a student defines oneself in relation to others highlights that different students experience a school context differently.

Beyond identity, the intensity of social interaction and judgment that students experience through the school’s contexts may heighten the impact of the hours spent in school on socioemotional and academic development beyond the formal curriculum. Taken together, the school community provides opportunities for the emergence of important and valuable social relationships, norms, and reciprocated obligations that build trust, shared information, goals, values, interests, and motivation among members of the communities. These social processes within schools are of interest to a wide range of scholars, including developmental psychologists, economists, sociologists, anthropologists, and education scientists. Through the emergence and maintenance of these social processes we understand how status hierarchies and opportunities to learn are structured and reinforced, contribute to the development of personality and motivation, and shape the cultures that emerge in schools (Coleman, 1988).

In all, the social relationships and culture that develop within the school may affect students’ learning, educational processes, educational attainment, adult earnings, and long-term health and well-being. As such, it may take on a larger role in the residential neighborhood or other collectives bound by the commonality of experiences. In addition to discussing the relevance for behavioral economics, Akerlof and Kranton (2002) provide an excellent description of the early studies about the school as a focal point of community and its role in identity development. From the standpoint of scholars of education and education policy makers, schools represent a major investment for every advanced and advancing society. The potential of school contexts to either amplify or undermine the investments made in curriculum, administration, teachers, and other personnel means that understanding the contexts of schools is a priority.

Also of interest to education researchers, policy makers, and public health professionals is that the social context of the school can be a setting in which antisocial development can occur. Recent events of school violence and bullying have heightened public awareness of the potential for rare but disastrous events to take place in schools. School fighting, substance abuse, negative body weight related pressures, and other health risk behaviors can both cause negative school climates and be a consequence of poorly functioning schools. These school safety issues stemming from the school’s context are important yet challenging to measure for a number of reasons. First, the negative outcomes that emerge from the context are relatively rare events. And second, we know very little about how such negative events come about or emerge from a school’s climate, or how to identify problem pockets within a school context. These challenges are complex; for example, efforts to disrupt a negative school environment, such as the use of metal detectors or visible mechanisms of social control or surveillance, may have their own negative consequences and exacerbate an already troubling situation (Arum, 2003).

### The Multiple Contexts of Each Student Within a School

Schools are situated in communities and are composed of students and their families, who may reside in diverse neighborhoods. Just as the school may provide a venue for defining community through shared activities of students and families, the larger community, district, state, and region can shape the social and curricular activities and priorities within the school by reflecting the surrounding social, cultural, economic and political contexts. Scholars from multiple disciplines have recognized the value of measuring these elements of school context. For example, school and neighborhood factors are independently associated with academic progress (Catsambis & Beveridge, 2001). Yet these relationships may be complex in that both absolute levels of neighborhood resources and the relative resources of a student’s neighborhood compared with others in the school predict educational attainment (Owens, 2010). The Moving to Opportunity study (Moving to Opportunity Program, 2015), a randomized experiment designed to estimate neighborhood effects, highlights the value of experimental data for establishing causal relationships between contexts and students’ outcomes.

### The School Contexts of a Student Over Time

Finally, the structure of schooling that groups similar age children together into classrooms, grade levels, and schools has implications for the study of school contextual effects on students’ experiences and outcomes over the course of childhood, adolescence, and into early adulthood. From preschool through college, the educational system structures changes each academic year such that groups of students and their school contexts are reconfigured. For example, the transition between middle school and high school often involves a building change that may be accompanied by merging of students from more than one school, as when several middle schools feed into a single high school or, alternately, a middle school splits between two or more high schools (Schiller, 1999). Students also transfer between schools at noninstitutionalized transitions (Sutton, Muller, & Langenkamp, 2013). The consequence of these changes—institutionalized and due to student or parental choice—is that students accumulate experiences in different contexts over the course of their school careers.

## Current Approaches to Measuring Context With NCES Data

The NCES has several programs that involve measuring school context: (a) a major new initiative, the School Climate Studies, and two notable and established programs, (b) the Secondary Longitudinal Studies Program, and (c) various data on schools and districts.

The School Survey on Crime and Safety (SSOCS) has collected information about crime at school and discipline from a sample of school principals beginning in 1999–2000 and every other year from 2004 to 2010. This survey has been used by researchers to study a range of factors related to how schools function for students, from how school crime is related to school composition and climate (Chen, 2008) to student achievement (Sulak, 2014). Replacing the SSOCS is the School Climate Survey Compendia (SCSC) of tools to measure school climate. The website safesupportivelearning.ed.gov describes the background and topics related to the initiative to measure “engagement, safety, and environment.” As part of the initiative, survey instruments are designed for middle and high school students, staff (teachers, leadership, other staff), and school administrators to administer as an information gathering step to improve school climate. The survey responses can be benchmarked to a national sample. For research purposes, the SCSC has the potential to make new and important contributions, but also has some serious potential limitations. On the positive side, the SCSC brings together many indicators of school climate in a single survey instrument to provide the opportunity to understand school climate in a more nuanced way through the study of covariation among indicators of reports from multiple perspectives (e.g., teachers, students, administrators). However, without adequate measurement of within school variation, achieved only with large enough probability samples of students in schools, the potential for understanding crucial sources of variation within a school will be lost. The value of measuring within school variation is discussed below. Measuring within school variation should be a high priority for developing an understanding of engagement and safety. In addition, the current plans do not include any capacity to link to administrative records, retain student identifiers for longitudinal data collection, or link student responses or climate measures to individual student outcomes. These shortcomings limit the potential value of the database if climate indicators cannot be observed in relation to diverse students’ outcomes.

The NCES Secondary Longitudinal Studies Program is a centerpiece of the portfolio for research on effects of school context on student development. It includes five studies: the National Longitudinal Study of the High School Class of 1972, the High School & Beyond (HS&B) longitudinal study of 1980, the National Education Longitudinal Study of 1988, the Education Longitudinal Study of 2002, and the High School Longitudinal Study of 2009. Each database has had a major impact on the research infrastructure and on policy; for example HS&B alone has provided data for at least 800 peer-reviewed articles, books, doctoral dissertations, and major reports (see HS&B, 2015, for a list). Together they allow researchers to study students’ academic progress over the life course and compare the processes across five decades. The emphasis of the program is on the academic development of students through high school and in the transition to adulthood. Unfortunately, these studies have limited capacity for analyzing within school heterogeneity. The studies are also limited in that they stop collecting data in early adulthood. The exception is a recent follow-up of the HS&B sophomore and senior panels (HS&B, 2015), which will allow researchers to study the long-run effects of school and education into midlife. Each study does include measures of school context from administrator reports on a range of topics (e.g., school attributes and resources, school climate, academic course offerings) and through the capacity to link school records to other school and district databases.

NCES collects information about schools and districts that provide rich indicators of school context. As with the SSOCS, these databases can be used to study climate, achievement, and other school factors related to context that are known to be important for students’ outcomes. An excellent example is Fiel’s (2013) analysis of trends in school resegregation. In contrast to the SSOCS, databases that cover all public schools or districts, such as those described below, are especially useful because they can be linked to the longitudinal studies of students, including those in the Secondary Longitudinal Studies Program (cf. Riegle-Crumb & Humphries, 2012) and local schools and districts (cf. Mickelson, 2015). Selected indicators have also been linked to the Adolescent Health and Academic Achievement Study (AHAA; cf. Langenkamp, 2010). Such approaches provide information for analysis of students’ exposure to multiple contexts over time.

The NCES and the Office of Civil Rights (OCR) sponsor four programs that collect data from every school and/or district in the United States. The Common Core of Data (CCD) program has collected and makes available data from every public school and local education agency (LEA), or school district, and state annually since 1986. The range of topics is extensive, covering information “about students and staff, including demographics; and fiscal data, including revenues and current expenditures” (http://nces.ed.gov/ccd/). Complementing these databases are indicators from the School District Demographic System, which compiles demographic and geographical information from the U.S. census products about persons residing within the attendance boundaries of LEAs. The School Attendance Boundary Information System is a similar product available at the school level for many public school attendance boundaries for limited years (2009–2012) (www.sabins-data.org). Information on the universe of private schools is collected every two years under the Private Schools Study (PSS) program. Together, the CCD and PSS cover the vast majority of schools in the United States.

Finally, the OCR Civil Rights Data Collection gathers information from every public school and LEA in the United States. These censuses, rather than samples, are collected only in selected years, with increasing frequency over the past decade. The purpose of the data collection is to provide information to help administer and enforce the civil rights statutes, so most programmatic indicators are disaggregated by race/ethnicity, sex, limited English proficiency, and disability (U.S. Department of Education, 2014).

As an increasing share of our population attends higher education, measuring postsecondary contexts is a priority in tracking students’ contexts over time. Since the 1960s, NCES has collected the Integrated Postsecondary Education Data System each year; it includes all institutions that participate in any federal student financial aid program. More recently, data from the National Student Clearinghouse have the potential to provide contextual data and unit record data for many individual students (Dynarski, Hemelt, & Hyman, 2015).

## Gaps and Areas for Improvement in Measurement

Many of the NCES school contextual databases described above include the same or similar repeated indicators over time to measure trends and to link to databases of individual students in schools. Such linkages provide much more analytic power for measuring school contextual effects over a students’ school career. Yet subcontexts within a school may exhibit considerable heterogeneity, and it is also possible to estimate heterogeneous school effects due to differences in how an individual experiences a context. Schoolwide indicators typically do not capture this heterogeneity.

Researchers have long recognized the importance of structural variations within schools for understanding students’ experiences and outcomes. For example, the practice of tracking to separate students into ability groups within a school was one of the first approaches to identify the powerful differences in opportunities to learn that could come about within schools (Gamoran, 1992; Gamoran & Mare, 1989). These unequal opportunities to learn were cause for concern (Oakes, 1985) and were followed by a call to detrack (Brewer, Rees, & Argys, 1995) such that few school administrators currently report that their schools track students. Yet many academic courses are still organized into sequences in which prerequisites lead to more advanced coursework. This is most common in mathematics and often results in students segregated into different courses, sometimes over the entire four years of high school (cf. Riegle-Crumb, 2006; Stevenson, Schiller, & Schneider, 1994). Similarly, sets of academic courses may be offered together, some in sequences and others as a result of scheduling constraints, resulting in effectively maintained inequalities in opportunities to learn within a single school (Lucas & Berends, 2002). Regardless of the exact definition of the differences in opportunities to learn, substantial evidence supports the claim that “second generation segregation” exists in many schools in the United States and results in inequalities in opportunities to learn (Mickelson, 2001).

Social and developmental psychologists and social network researchers have recognized that the tendency of people to form friendships according to homophily, or similarity of attributes, such as race, may shape peer contexts within a school (Moody, 2001). These friendship groups may exert powerful normative influences on students’ behavior and identity. Differences in adolescent students’ identity have long been recognized by researchers, and may even be characterized by their perceived connections to or position in the school (e.g., Barber et al., 2001; Coleman, 1961; Willis, 1977). Students may fine-tune their reference group to suit their self-perceptions and identity (Mueller, Pearson, Muller, Frank, & Turner, 2010), with consequences that must be understood in terms of the fit between the student and his or her context within the school. It is worth noting that this fit may have positive, prosocial consequences (Riegle-Crumb, Farkas, & Muller, 2006) or negative effects for adolescents who do not fit in (Wilkinson & Pearson, 2009), or place some students in contexts that promote negative behaviors (e.g., drinking—Crosnoe, Muller, & Frank, 2004; suicide ideation—Abrutyn and Mueller 2014). Each of these approaches underscores the value of recognizing heterogeneity within schools and how students’ attributes interact with the context to shape their experiences and outcomes.

The heterogeneity of school contexts presents measurement challenges because (a) students are members of different contexts, in and out of schools and over time; (b) each school has a unique internal structure; (c) within a single school, different students may experience a similar structural position differently; and (d) schools include multiple actors—students, teachers, staff, and administrators—as well as parents and possibly community leaders, each of whom will have a unique and potentially valuable perspective. Ideally, any procedure to estimate the context would be empirically derived using data from the school and people within the school (students as well as other actors) so that the measures tap the uniqueness of the school and can be linked to students and other actors in the school and compared across schools. The recent events of school violence stemming from students’ alienation underscore the value of recognizing that students may occupy different social positions in the school, and may experience the school and its contexts very differently. Instances of bullying or other forms of school violence may come about when a student becomes alienated and feels like an outsider, in part because of the (perceived) insiders in the school. A single school may have both insiders and outsiders within the same contextual spaces. It is only by analyzing the relationship between the internal structure of schools and the individual students within those spaces that we will be able to identify and understand how some schools might at once promote learning for some students and become unsafe spaces for other students.

To date, the NCES approach to measuring school context is strong for identifying nationally representative samples of schools and students and for evaluating trends over time. The extant school contextual databases measure some aspects of the structural elements within schools for analyzing between school variation, but they lack important detail. None of these surveys adequately measures the contextual heterogeneity *within* schools, nor can the different contexts in the school be linked to particular students in the school. Doing so is a crucial first step toward developing policies that serve the diverse needs of our nation’s students. Enhancing our understanding of how school policies can impact structures of opportunities within schools for different students is an important priority. Data collection to understand how policies shape positive school contexts requires the capacity to measure within school contexts over time and would ideally allow for the estimation of effects of policy interventions.

## New Opportunities for Measuring School Contexts

New approaches for measuring school contexts can capitalize on advances in technology and data availability to better capture the heterogeneity within schools and link context(s) to individual students. With the ability to link contexts to individual student data, such as that gathered through interviews or for administrative purposes, we can understand how the student experiences his or her context(s). These new data opportunities have the capacity to provide rich empirical evidence at multiple levels to improve schools. This section begins by discussing network approaches for characterizing the structure of relations among students, then elaborates on measuring heterogeneity among students, describes the potential for considering multiple perspectives that include teachers, administrators, and parents, and concludes the section with a brief discussion of the implications of the advances for more strategic sampling.

Network methods offer promising tools for identifying clusters of students who share courses. A two-mode approach (Field, Frank, Schiller, Riegle-Crumb, & Muller, 2006) was developed using data from the National Longitudinal Study of Adolescent Health (Add Health). The longitudinal component of Add Health includes approximately 200 students per school and data from AHAA, which collected and coded their high school transcripts. The two-mode approach detects emergent clusters, called “local positions,” of students who take sets of courses together. Each school has a unique structure that represents a meso-level context, or set of local positions, in the school. These tap a source of within school heterogeneity that is more nuanced than tracks or sequence of courses. Local positions are defined by a set of courses that typically contain fewer students than a track, and they are derived empirically and are unique to each school. Attributes of students in the local position predict whether the student invests in demanding math courses in subsequent years (Frank et al., 2008), and the friendships that form in the later years of high school as some students prepare for postsecondary study (Frank, Muller, & Mueller, 2013).

Other network approaches using similar or enhanced data from administrative records or from teachers may offer important advances as well. Add Health did not contain information from teachers about themselves, their perspectives on their students, or what courses they taught. Indeed, it did not even identify which teachers taught the courses that were associated with the positions. Such data, which are possible to obtain from administrative records, would almost certainly provide an empirical foundation for further advances in estimating school structures, students’ positions in them, and the learning opportunities and social experiences of students in schools.

A comprehensive mapping of the within school structure as defined by the everyday lives of the students, such as that achieved with the local positions, can place a focal student in a specific context within a school. With information derived from representative samples of students in each school, the attitudes and behaviors of a focal student’s peers, such as in the local position, can be used to estimate peer effects. For example, it would be possible to develop nuanced models for the role of peers in adolescent girls’ progress in STEM fields. Incorporating information from teachers or even parents of the students could allow the researcher to measure or triangulate climate-related reports from multiple perspectives within the context or local position. Similarly, characteristics of the other students in a position could be based on indicators from administrative records (e.g., test scores, grades) and used to estimate contextual effects for different students (e.g., girls and boys, academically high- or low-performing students).

Administrative records of students’ addresses can provide information about the neighborhoods of students who attend the school. Beyond measuring aspects of social class (e.g., housing values, whether housing units are owned or rented, unemployment rates), neighborhoods vary in their crime and arrest rates, type of crime, adult (and parent) incarceration, the treatment of minors as adults in the criminal justice system, racial segregation, and other features of social organization that may have consequences for the everyday lives of students and for the functioning of the school. In addition to providing enhanced information about students’ peers, such information would be valuable for comparing schools, for example, charter schools to comprehensive public high schools.

Other sources of data may provide rich portraits of students’ lives. Medical records could provide information on child health, which is very poorly measured in most studies that do not explicitly focus on the topic. Information on parents’ employment, available from state or federal data systems, would also greatly enhance our understanding of students and their peers. And parents’ income tax records linked to student records make it possible to more accurately measure important concepts such as value-added by teachers and schools (Chetty, Friedman, & Rockoff, 2014).

New and rapidly changing technologies have powerful implications for gathering rich information about how students (and teachers and parents) live their lives. For instance, methods such as experience sampling methodology (ESM), which queries students about what they are doing and how they are feeling in the contexts of their lives throughout the day, are improving with technological advances and have potential for education research (Zirkel, Garcia, & Murphy, 2015). Moments in time are sampled and can be analyzed to estimate, for example, the level of challenge a student feels when engaging in activities in classrooms or at home watching television or doing homework (for description of the method, see Hektner, Schmidt, & Csikszentmikalyi, 2007; Moneta, 2012). Newer technologies have made possible the collection of biomarkers to gauge reactions to contexts or record real time behavior, such as activity level. Large amounts of data on biomarkers, such as heart rate and exercise, are collected with devices like Fitbit and are used in marketing; they could also provide a wealth of data for academic research, particularly if used in conjunction with techniques such as ESM. And students (or other actors) can be tracked using global positioning system (GPS) technology to place them in geographic proximity to one another, or to otherwise provide a more accurate and less intrusive strategy for placing them in contexts. Students also leave artifacts of their feelings and relationships online (e.g., Facebook, Twitter), which could be coded and analyzed. Nonetheless, indicators of attitudes, feelings, and perceptions of students, teachers, parents, and significant others are still valuable—possibly even more valuable with the supporting location and biomarker data—and must still be acquired from self-reports. These data from new technologies will likely spur advances in methodology, and also produce a much richer understanding of what happens in schools.

Along with rapidly changing technology are heated political debates about whether and how data artifacts from our daily lives should be maintained, retained, and used. What is technically possible may be politically complicated, and both landscapes are changing rapidly. For any new study design it would be helpful to work with experts who have not traditionally consulted on designs of education and social science databases, for example, technology experts from Silicon Valley or persons involved in marketing or analysis of big data who are already familiar with methods for compiling data into useable forms. These recommendations are part of a larger conversation about the potential to update indicators at relatively low cost with high return (Davidson, 2015). The point is that with the methodology in place to measure both school context and within school contexts—linking the individual student to contexts—rich possibilities to assess how schools impact students become possible.

Obtaining reports from multiple actors in a school is an additional source of heterogeneity that has proven valuable for tapping school context. The secondary longitudinal studies have parent components, teacher reports on sampled students, and administrator reports. Approaches to gathering data about multiple perspectives using multiple modes for collection was refined on a large scale for the Measures of Effective Teaching study (Measures of Effective Teaching Project, 2014). Another notable application of measuring context through perspectives of multiple actors was employed in an implementation study of a program designed to increase social capital in schools (Gamoran, Turley, Turner, & Fish et al., 2012). Because the program was designed to improve the social context of the school, it was imperative to measure the context from the perspectives of all actors in the school. Furthermore, this approach allowed the researchers to gauge effects of context for diverse students.

New data sources also open up new possibilities for sampling. NCES and others have long used the CCD, PSS, and the Quality Education Data as sampling frames for two-stage designs that first sample schools and then samples students within schools from school rosters. In theory, the state administrative data systems could provide a much richer portrait of schools, their contexts, and students in the context. These censuses of schools and students in them can provide a sampling frame by providing indicators of structural features of the schools and variation between and within them. A limitation of state data systems is that they only include public schools. Using this information should make it possible to draw samples that are more closely tailored to the purposes of a study, and especially to provide information relevant for targeting oversamples. For example, state data systems provide rich information about students’ learning and growth in learning, how students move and transition between schools, the courses that they take, and attributes of teachers, all by students’ race and ethnicity, gender, and age. These possible indicators of opportunity to learn, school quality, and other aspects of the school process and context could be used in sample selection.

## Special Considerations in Measurement

As suggested above, measuring the effects of school contexts is not only valuable but is best accomplished by considering the interaction of the contexts with the individual. Several additional issues are worth consideration.

First, a well-recognized source of variation in how a particular student responds to a context concerns the student’s age and developmental stage. Barber et al. (2001) found that the stage-environment fit varies along racial and ethnic lines. As students age they are better able to handle more complex social arrangements, but this pattern also varies according to race and ethnicity, with African American students benefiting longer from the less complex social settings. A good example is in the transition from elementary to middle school, which for many students means transitioning to larger classes and classes taught by many different teachers. Although such an arrangement allows students to be taught by teachers with more specialized academic knowledge, it also places higher demands on the student to navigate more relationships and possibly more complex relationships with teachers who may form more superficial impressions of students if they have fewer opportunities to get to know them as individuals. African American students may face greater risk in navigating these transitions, at least in some settings. Thus, in measuring the context it is important to take into account the age, developmental stage, or other characteristics that might shape the way a student experiences a particular context.

It is also important to recognize that students select into contexts, and they are members of multiple contexts, in different spaces and across time. A failure to properly measure the selection or the effects of unmeasured contexts can result in the mismeasurement of the effects of a focal context. Although a full discussion of these measurement challenges is beyond the scope of this article, data generated from non-experimentally designed collections are better suited to descriptive aims, theory building, and hypothesis generation, and are not adequate to determine a causal effect of a context on individual outcomes. It is especially important to remember that randomized experimental design is better suited to test the potential effect of a particular policy initiative.

## Priorities for Future Measurement of School Contexts

The purpose of this article is to discuss approaches to measuring school contexts as they relate to students’ experiences, development and outcomes, broadly defined. Structural features of the school context have important implications for students, yet they are often poorly measured in large-scale national studies. Rapid changes in technology and big data along with new methodologies for collecting and analyzing data, contemporary challenges to gaining cooperation for conventional data collection, and the changing nature of students’ lives must all be considered in assessing priorities for future data collection. These technological advances and new challenges suggest that there would be a substantial benefit from rethinking the traditional models of data collection and analysis for measuring context. The following priorities take these considerations into account:

1. Collect sufficient data about students within a school to systematically measure within school heterogeneity and to make it possible to locate a focal student’s position relative to other students in the school.
2. Collect data from other members of the school community, for example teachers, counselors, and parents, such that their data can be (a) linked to focal students and (b) used to characterize the subcontexts within the school.
3. Rethink what information is necessary to collect directly from students and other members of the school community and what information can be collected through passive approaches. Information about how students feel about themselves and others, their identity, interests, moral and social development, and expectations and hopes for the future is important for designing successful education policy, and the student most likely must directly report this type of information. Yet, new technology has resulted in an ever-changing landscape for possibilities and costs of data collection and study design. Each new data initiative should reevaluate what is possible.
4. Examine the potential of collecting and processing nontraditional sources of data, including but not limited to administrative records from various sources. These data may extend to nonschool contextual settings for the student, such as family or out-of-school activity, and to postsecondary and adult enrollment in schools, courses, and training.
5. Explore innovative approaches to survey methods that use newly available technology and possibly involve passive cooperation of respondents, for example using wearable technology or administrative data.
